# The Princess and the First Thousand

**Published:** 1890-07-12

**Authors:** 


### The Hospital

A Weekly Journal of

### Science, Medicine, Nursing, and Philanthropy.

*For Hospitals, Asylums, Medical Practitioners, Students, Nurses, Families, Parishes, Congregations, and the Charitable Public.*

Vol. VIII.—No. 198.] [July 12, 1890.

### THE PRINCESS AND THE FIRST THOUSAND.

It is often said and believed that wealth is the surest winner in the race for public honours. Cynicism finds its pleasure, such as it is, in following this line of thought and in compelling attention to it. But in this country, and no doubt universally, there are ways to honour which do not pass through the avenues of wealth. Those five or six hundred nurses who were welcomed and smiled upon by the Princess of Wales at Marlborough House, on Friday week, were probably as poorly endowed with worldly goods as any five or six hundred respectable women could well be. Yet one of the noblest princesses of her time, and the sweetest woman, made those nurses free of her generous hospitality and received them as a sister would. The sight was more than pleasing. It was better than "in the brave days of old, when the rich man helped the poor man, and the poor man loved the great." It was better, because the nurses were not there to receive the alms or the bounty of the princess, but to be encouraged and stimulated along the honourable pathway of self-help.

Nurses have for some time received a larger share of public attention than any other women of their own class. Is this a purely artificial state of things? Or is there something about nurses, distinguishing them from other women, which will maintain them at their present elevation in public regard? A critical spectator at the Merchant Taylors' gathering and at Marlborough House, could not but be conscious of certain peculiarities about the nurses which were sufficiently marked to differentiate them from all other women of their own age and condition in life. Two characteristics were plainly distinguishable: discipline, and readiness of resource. The very way in which their uniforms were worn showed training; and the promptitude with which they fell into order and line proved how thoroughly experienced they were in the art of quick and intelligent obedience. It is plain that the three or four years of hospital life which most nurses have to pass through, do not merely make nursing experts of them, but give them general culture of a very valuable kind. Many a nurse who has had poor opportunities of school education before commencing her training, leaves the hospital with a cultivated mind and with practical intelligence much above the average of her sex. All this, and more was clearly evident to the sympathising spectator at Merchant Taylors' Hall and at Marlborough House.

The just man likes to see every person receive his due. In a sense, nurses receive their due and sometimes perhaps a little more; but in other particulars they receive very much less. Wages ranging between twenty and thirty-five pounds a-year cannot be said to be adequate pay for well-trained nurses, even though supplemented by one or two uniforms. At the same time, the supply of candidates for nursing appointments is so abundant that it would be futile to expect any considerable increase of pay. It speaks well for the sense of justice that dominates our fellow countrymen that they are so universally anxious to make amends to nurses in other ways. The National Pension Fund had its origin in a sense of justice. "A fellow feeling makes us wondrous kind"; and to a hospital worker, who has known by stern experience the limited opportunities of hospital officials, was due the inception of what has since become a great and widespread popular movement. But the Pension Fund could have had no chance of permanent existence and growth if it had not found itself planted in a soil prepared for it. For a quarter of a century the public and the medical profession have been finding out the incalculable value of trained nurses. There is ample evidence to show that the starting of the Pension Fund came as a positive relief to the minds of many persons whose lives had been saved or their health restored mainly through the agency of sick nurses. To those persons an opportunity was offered for the first time of doing something practical to show their appreciation and gratitude. The fact that a Bonus Fund of £40,000 has been raised within three years, and that largely by the efforts of one individual, is proof positive of the firm hold that nurses have taken upon the regard and affection of those whom they have served. When to this material reward there is added the warm recognition of their services and the public espousal of their cause by those who are so near the throne of this realm as their Royal Highnesses the Prince and Princess of Wales, the nurses cannot but feel that there is a sincere desire among all ranks to prove to them how grateful a British public can be.

The Princess of Wales put the top-stone and crown upon what has been a very arduous undertaking. The National Pension Fund for Nurses was, at its commencement, the breaking of entirely new ground. No such association had ever been created before; and there was consequently neither experience to guide nor example to warn of possible danger. Entirely satisfactory, not to say triumphant, as were the proceedings at Marlborough House on Friday week, they were no index of the arduous labours, the deep anxieties, and the alternate hopes and disappointments of three strenuous and fighting years. At the outset the nurses were drawn in different directions by critics who had no just grounds for their opposition, and who made up for the want of justice in their cause by unscrupulousness in its maintenance. Of course those who knew anything whatever about the nature of the business in hand understood quite well the real cause of the opposition. But nurses did not and could not be expected to understand it. At one time it seemed possible that the £20,000 of the four city merchants would actually be flung back in their faces as a despised and worthless gift. But inexperienced as the majority of the nurses were in the ways and arts of disappointed and defeated persons they had practical good sense enough to know that men who were willing to give £20,000 for their benefit were at least as likely to be their friends as those who had nothing to give them, and were only anxious to take something from them if possible.

But the fighting days are over, and the National Pension Fund for Nurses is now, as the Prince of Wales said, in the fullest sense of the word a permanent "national" institution. His Royal Highness briefly sketched the history of the Fund from the date of its birth to the present time. He announced that nurses had themselves collected a sum of £2,200 to found a Junius S. Morgan Benevolent Fund. He paid a passing tribute to the memory of Mr. Morgan, who, he said, had been the generous donor of £10,000. He commended specially those nurses who had, unaided, joined the Fund to the number of several hundreds, and stated that a sum of £5,000 had been set apart by the managers for the special purpose of the augmentation of their pensions. He expressed the conviction that the Pension Fund was the very kind of thing that nurses needed, and that all nurses of every class should hasten to avail themselves of its advantages.

As warmly as the Prince of Wales spoke Mr. Walter Burns, of the work that had been done by Mr. Morgan and the other City merchants, and of the work that Mr. Morgan's family yet intended to do. "Nothing succeeds like success," and the Pension Fund has been successful beyond the most sanguine hopes of its founders. Whilst, however, it is impossible for the Fund to fail, it is quite possible for many nurses to fail in securing its benefits. No fund can help those who will not make an effort to help themselves. We have reason to know that the managers are anxious to meet halfway those who can do the very least in their own behalf provided they will make some sort of attempt to do that least. Those who sit still and will not so much as write a letter of inquiry must not be surprised when old age comes if they find their sister nurses in plenty and themselves in poverty. In this world, it is true, that to her that hath shall be given, and she shall have abundance, whilst from her that hath not and maketh no effort to gain, shall be taken away even that which she seemeth to have.

